# Lung cancer: a 6-field technique using lateral beams in conformal radiotherapy for bilateral supraclavicular lymph node metastases

**DOI:** 10.1186/2193-1801-3-733

**Published:** 2014-12-13

**Authors:** Shinichi Tsutsumi, Takuhito Tada, Tomoko Maekado, Masahiro Tokunaga, Noriko Tanaka, Ai Kobayashi, Eiichiro Okazaki, Shougo Matsuda, Masako N Hosono, Yukio Miki

**Affiliations:** Department of Radiation Oncology, Osaka City University Graduate School of Medicine, 1-4-3, Asahimachi, Abeno-ku, Osaka, 5458585 Japan; Department of Radiology, Izumi Municipal Hospital, 4-10-10, Fuchucho, Izumi, 5940071 Japan; Department of Radiology, JCHO Osaka Hospital, 4-2-78, Fukushima, Fukushima-ku, Osaka, 5530003 Japan

**Keywords:** Lung cancer, Conformal radiotherapy, Supraclavicular lymph node metastasis

## Abstract

A 6-field technique using lateral beams in conformal radiotherapy was developed for patients with bilateral supraclavicular lymph node metastasis of lung cancer. The possibility of using this technique in practice was evaluated. Six fields with the same isocenter point (IP) were arranged. Two fields using anterior-posterior opposed beams involved all of the planning target volume (PTV). The next 2 fields using off-cord oblique beams involved the PTV inferior to the IP. The remaining 2 fields using lateral opposed beams, that shielded the spinal cord, involved the PTV superior to the IP. The oblique 2 fields and lateral 2 fields were connected using a half-beam technique. In 6 patients with non-small-cell lung cancer (NSCLC, n = 4) or small-cell lung cancer (SCLC, n = 2), treatment re-planning based on this technique was performed. This technique was applicable in 4 patients with NSCLC, in whom the general criteria of radiotherapy for lung cancer were met. In 2 patients with SCLC, the cumulative volume of lung that received more than 20 Gy exceeded 37% of the total lung volume. This technique was usable in 67% of the patients and was not necessarily contraindicated in the other 33%.

**Electronic supplementary material:**

The online version of this article (doi:10.1186/2193-1801-3-733) contains supplementary material, which is available to authorized users.

## Introduction

The standard of care for patients with locally advanced lung cancer is concurrent chemo-radiotherapy. By using three-dimensional conformal radiotherapy (3D-CRT), a more conformal dose distribution to the target volume is obtainable, and the dose administered to normal tissue is reduced (Hayman et al. 2001; Bradley et al. 2002; Rosenzweig et al. 2000; Anscher et al. 2002). To reduce the dose to the spinal cord, off-cord (i.e., the spinal cord is outside the field) oblique beams are used.

In two-dimensional radiotherapy or 3D-CRT, when there are bilateral supraclavicular lymph node metastases, simple fields using off-cord oblique beams cannot be used. Usually, each side of each supraclavicular lymph node is irradiated separately after the initial field irradiation using anterior-posterior opposed beams. However, the conformity of the dose distribution is not sufficient when simple anterior-posterior opposed beams are used. And the radiation field becomes complex in the later part.

To improve the conformity, a 6-field technique using lateral beams was developed. In this study, the possibility of using this technique in practice was evaluated by performing re-planning in prior patients.

## Materials and methods

The protocol for this study was approved by the institutional review board of Izumi Municipal Hospital. Patient informed consent for this study was not obtained because the practical treatment had already finished and only dry run was performed in this dosimetric study.

### A 6-field technique

**Figure 1:**
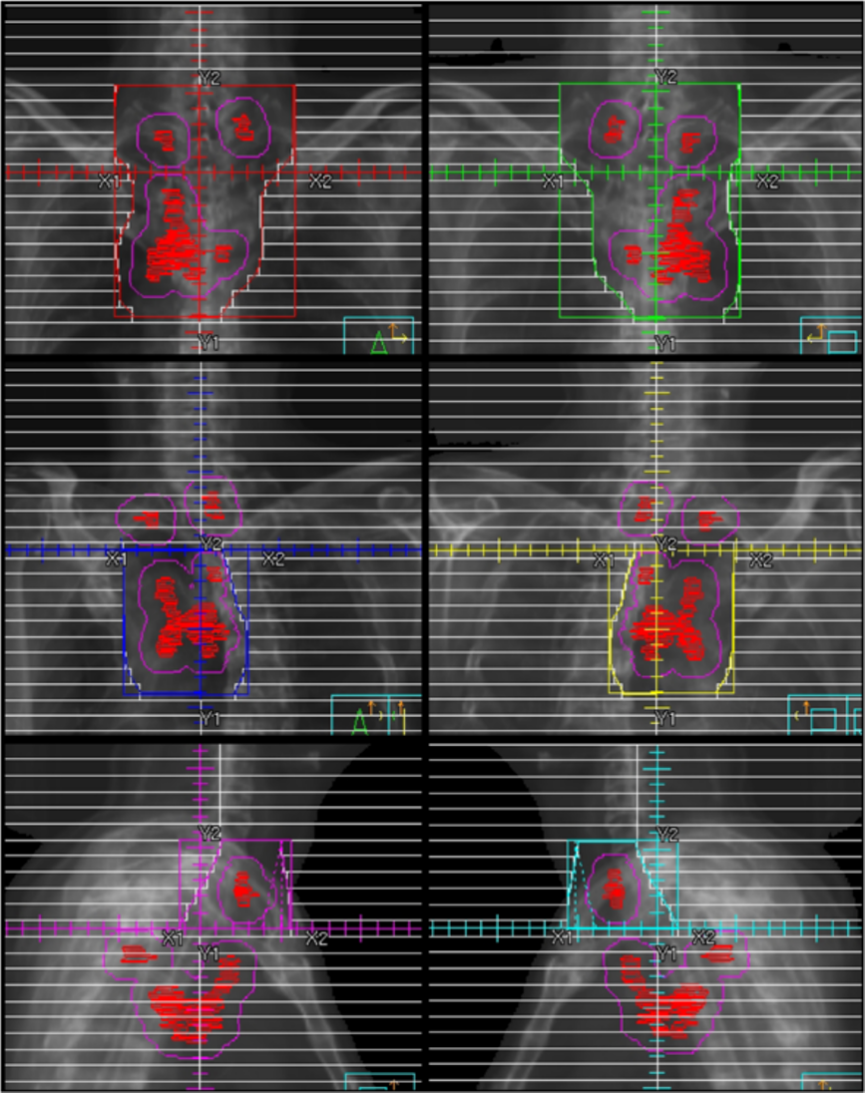
**Beams’-eye-view images show an example of the 6-field technique.** All 6 fields had the same isocenter point.

To perform 3D-CRT, 6 fields were arranged. All 6 fields had the same isocenter point (IP). Two fields using anterior-posterior opposed beams involved all of the planning target volume (PTV). The next 2 fields using off-cord oblique beams involved the PTV inferior to the IP. The remaining 2 fields using lateral opposed beams involved the PTV superior to the IP. The oblique 2 fields and lateral 2 fields were connected using a half-beam technique (Figure 1).

### Patients

Between July 2005 and March 2013, a total of 6 patients with bilateral supraclavicular lymph node metastases of non-small-cell lung cancer (NSCLC, n = 4) or small-cell lung cancer (SCLC, n = 2), underwent definitive radiation therapy using previous planning without the 6-field technique at our hospital. All patients were clinical stage IIIB. The tumor characteristics are summarized in Table 1. The treatment-planning CT data of these 6 patients were used for this dosimetric study.

**Table 1:** **Tumor characteristics**

Patient number	Location (lobe)	Size	Positive nodes (#)
1	Rt. lower	42 mm	2R, 4RL, 7, 10
2*	Rt. upper	56 mm	2RL, 3a, 4RL, 7, 10
3	Rt. upper	23 mm	3p, 4R, 7, 10
4*	Lt. lower	62 mm	2R, 3a, 4RL, 7, 10
5	Lt. upper	36 mm	2L, 4L
6	Lt. upper	95 mm	4RL, 5, 6, 7, 10

Size: long axis measurement.

*Small cell lung cancer.

### Treatment re-planning

A commercial treatment planning system (Pinnacle3 version 9.2, Philips Medical Systems, Bothell, WA, USA) was used to generate treatment plans. The volumetric treatment-planning CT data, which were used for the previous planning, were re-input into the system. A 2-mm slice thickness was used in all patients.

In this study, elective nodal volumes were not included within the PTV. The gross tumor volume (GTV) was defined as the volume occupied by visible disease. The GTV included the primary tumor and the involved lymph nodes measuring larger than 1.0 cm (short axis measurement) or lymph nodes with a diameter of 5 mm or more shown by positron emission tomography. The clinical target volume (CTV) was defined as the GTV plus a margin of 5 mm for all borders. The PTV was the CTV plus a margin of 5 mm or more. A part of the margin for the PTV could be reduced to shield the spinal cord.

Tissue inhomogeneity corrections were used. For beam energy, 6-10 MV was to be used. The prescribed dose was 60 Gy in 30 fractions. The normalization of the treatment plan covered 95% of the PTV with the prescribed dose. A contiguous volume of no more than 2 cc inside the PTV exceeded 20% of the prescribed dose. The cumulative volume of lung that received more than 20 Gy (V20) should not exceed 37% of the total lung volume, and the maximum dose to the spinal cord should not exceed 50.5 Gy. Lung dose constraints were based on a previous report (Bradley et al. 2005).

## Results

**Figure 2:**
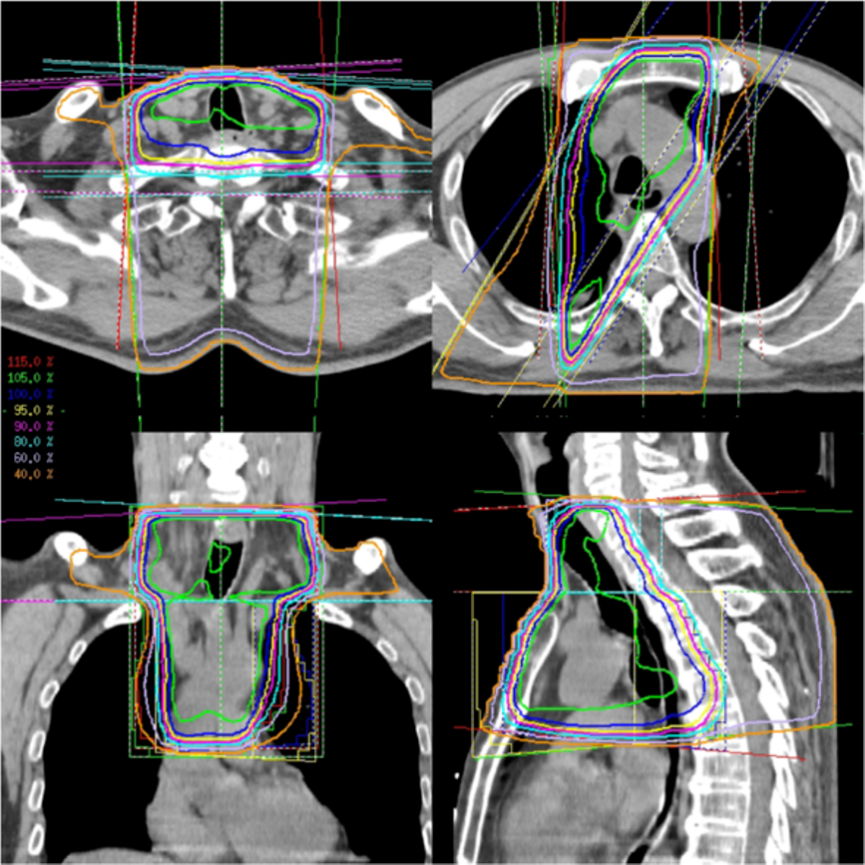
**At the border between the oblique beams and the lateral beams, no areas showing over- or under-dosing were observed.** A high dose area was observed at the anterior part of the neck. However, this area did not exceed 20% of the prescribed dose.

In all 6 patients, the 6 fields contained the PTV. The V20 of the lung and the maximum doses are summarized in Table 2. In 2 patients with SCLC (Patients 2 and 4), V20 of the lung exceeded 37%. At the border between the oblique beams and the lateral beams, no areas showing over- or under-dosing were observed (Figure 2). A high dose area was usually observed at the anterior part of the neck (Figure 2). However, this area did not exceed 20% of the prescribed dose. Although Patient 2 and Patient 4 had high V20 of the lung, all the criteria were met in the remaining 4 patients.

**Figure 3:**
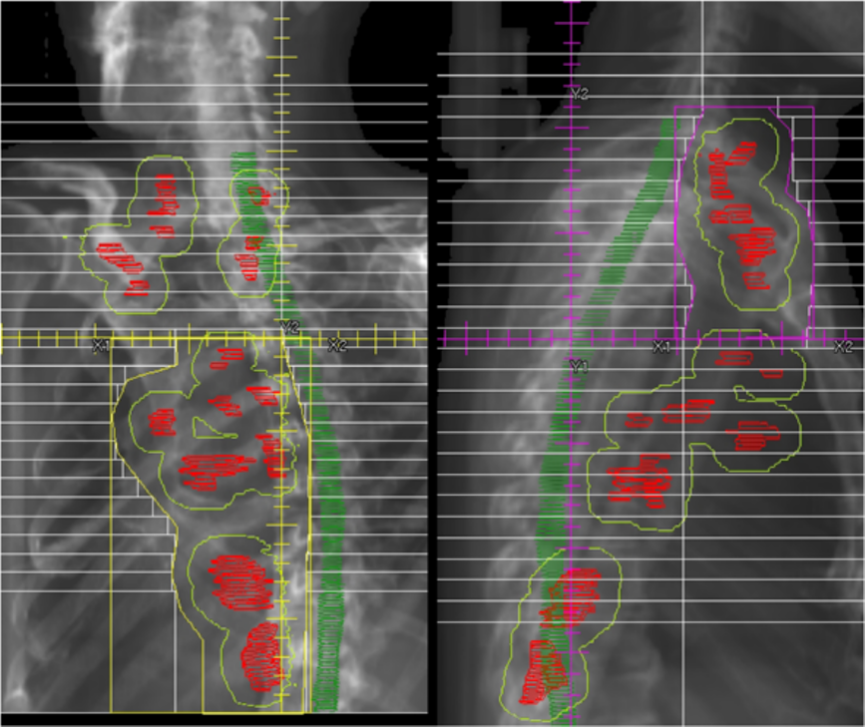
**In Patient 4, the isocenter point had to be shifted to the inferior-posterior direction, because the jaw capacity of the linear accelerator was 20 cm.** Therefore, the lateral beams contained more of the normal lung volume.

In Patient 4, the primary tumor was located at the posterior-inferior area of the lung. Because the jaw capacity of the linear accelerator was 20 cm in our institution, the IP had to be shifted to the inferior-posterior direction to contain the lower margin of the PTV (Figure 3). Therefore, the lateral beams contained more of the normal lung volume, and the V20 increased.

**Table 2:** **The V20 of the lung and the maximum doses**

Patient number	V20 (lung)	Dmax (spinal cord)	Dmax (radiation field)
1	35%	50.1 Gy	68.2 Gy
2*	47%	49.4 Gy	69.5 Gy
3	18%	45.4 Gy	68.1 Gy
4*	43%	46.2 Gy	71.5 Gy
5	9%	50.1 Gy	70.7 Gy
6	36%	47.8 Gy	68.0 Gy

V20 = The volume of the organ that received more than 20 Gy. Dmax = The maximum dose.

*Small cell lung cancer.

## Discussion

Radiation therapy for patients with bilateral supraclavicular lymph node metastases has long presented a challenging anatomic problem. Several techniques to irradiate the supraclavicular lymph nodes in breast cancer or head-and-neck cancer have been reported (Scrimger et al. 2000; Lu et al. 2003; Madu et al. 2001; Dogan et al. 2007; Gielda et al. 2011; Yang et al. 2011; Duan et al. 2004). However, similar techniques for lung cancer do not exist. Additionally, no reports have presented the use of the lateral beams for the supraclavicular lymph nodes. Arrangement of radiation fields using a half-beam technique is not novel, but it is not easy to use of lateral beams for the supraclavicular lymph nodes since the difference in thickness between the shoulder and the neck is very large. To introduce the possibility of their use is one of the aims of this report.

When the 6-field technique is usable, several benefits are estimated compared the classical method. Because both sides of supraclavicular lymph nodes are irradiated at the same time, treatment planning is not complicated and the actual irradiation is convenient. Furthermore, the reduction of toxicity is obtainable due to the improved conformity compared to the classical method. In the classical method, anterior-posterior opposed beams and off-cord oblique beams are sequentially used. Thus, considerable volumes receive a fractional dose of 2 Gy outside the PTV. On the other hand, these beams are concurrently used in conformal radiotherapy and the only volumes irradiated by all beams receive the fractional dose of 2 Gy. In the other volumes, the fractional doses are balanced. Therefore, when the same dose distribution in the total dose of 60 Gy is obtained, conformal radiotherapy with the balanced fractional doses is less toxic than the classical method.

The 4 patients with NSCLC met the criteria for the 6-field technique. In the 2 patients with SCLC, when re-planning was performed using a dose of 45 Gy in 30 fractions (accelerated hyperfractionation), V20 of the lung was 43% and 37% for the Patient 2 and the Patient 4, respectively. Even in the treatment of SCLC, the involved field is usable based on recent reports (Shirvani et al. 2012; Han et al. 2012; Xia et al. 2012). The same limit of the V20 of the lung was used because pulmonary toxicity has not increased when accelerated hyperfractionation has been used (Tsujino et al. 2006). Therefore, this technique is considered usable for patients like Patient 4. Even in patients like Patient 2, this technique may be usable when the margin for the PTV is decreased and/or the radiation dose is modified. In the patients who can receive radiotherapy using the classical method, we estimate that there is a high probability that this technique is appropriate.

Several limitations were observed for this technique. The location of the primary tumor limited treatment planning. The lateral beams for the supraclavicular lymph nodes are unable to contain the lung behind the spinal cord. When the PTV for the primary tumor is in this location, it is contraindicative. In addition, the IP has to be established within the jaw capacity. When the primary tumor is located at the inferior area of the lung, shifting of the IP from the proper position is necessary, which unfavorably influences the V20 of the lung. Furthermore, high-dose areas were observed at the anterior part of the neck due to the large thickness difference between the shoulder and the neck. However, the high dose areas were at a distance from the brachial plexus. When the dose is too high, it is necessary to reduce the weight of the doses of the lateral beams or to use the field-in-field technique.

When intensity-modulated radiotherapy (IMRT) is available, the problem of poor conformity in the classical method is solved. However, IMRT is not available everywhere. In addition, IMRT is much more expensive than 3D-CRT, and the field size is limited in several IMRT machines. Furthermore, IMRT sometimes increases the volumes of low doses for thoracic normal tissue and that can be associated with injury to lung tissue (Murshed et al. 2004; Liu et al. 2004; Yorke et al. 2005; Chapet et al. 2006). The addition of the option to use the 6-field technique is not necessarily useless even in the institutions where IMRT is available.

Patients with bilateral supraclavicular lymph node metastasis who are candidates for definitive radiotherapy are relatively rare. In our institute, they were less than 3% of all patients with lung cancer who underwent radiotherapy. Therefore, patients with SCLC were added in the present study.

Although data for more patients is required for proper analysis, it takes long time in data accumulation due to the rareness of the disease. Since the 6-field technique is usable in some patient group at the least, early introduction of this technique is preferred.

Recently, the 6-field technique was used in our clinic for 2patients with lung cancer, in whom planning and irradiation were satisfactorily performed. Further evaluation is needed to determine its clinical benefits and limitations.

## Authors’ original submitted files for images

Below are the links to the authors’ original submitted files for images. Authors’ original file for figure 1 Authors’ original file for figure 2 Authors’ original file for figure 3
