# Supplementary figures and images for: Lung cancer: a 6-field technique using lateral beams in conformal radiotherapy for bilateral supraclavicular lymph node metastases
Source: Springerplus. 2014 Dec 13;3:733. doi: 10.1186/2193-1801-3-733 (PMC4320133; doi:10.1186/2193-1801-3-733)

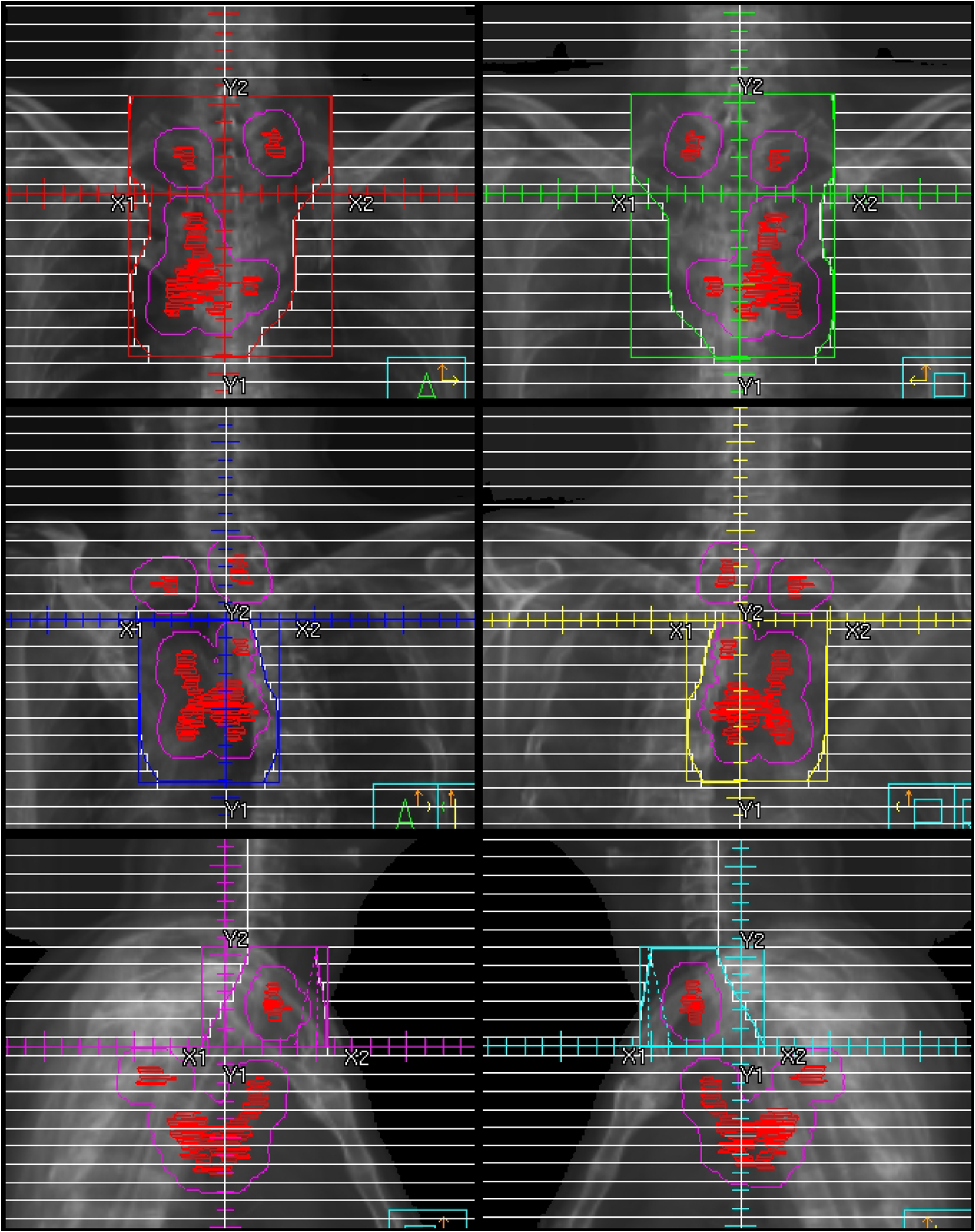

Supplement: Supplementary file 1 — Authors’ original file for figure 1 [file 40064_2014_1486_MOESM1_ESM.tif]

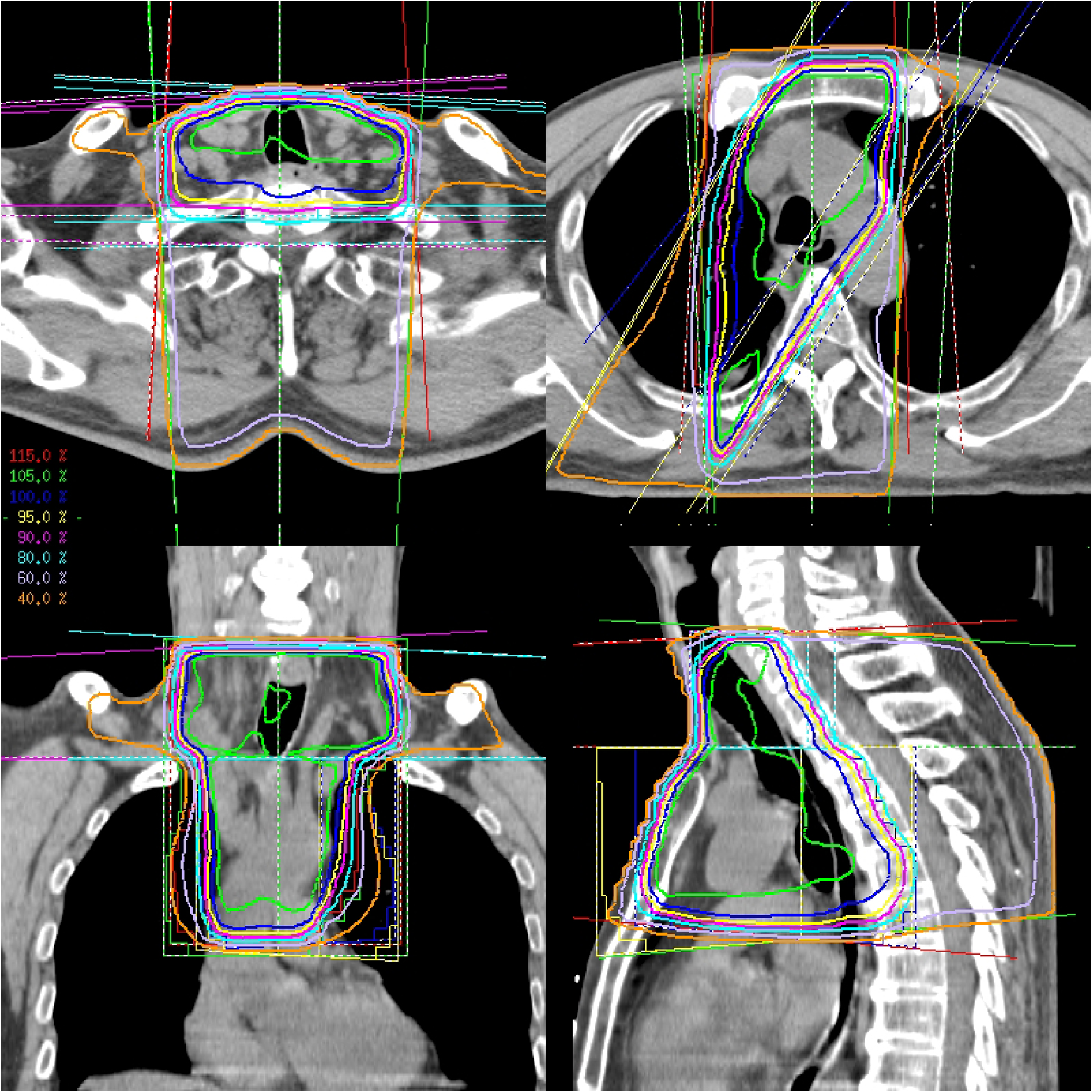

Supplement: Supplementary file 2 — Authors’ original file for figure 2 [file 40064_2014_1486_MOESM2_ESM.tif]

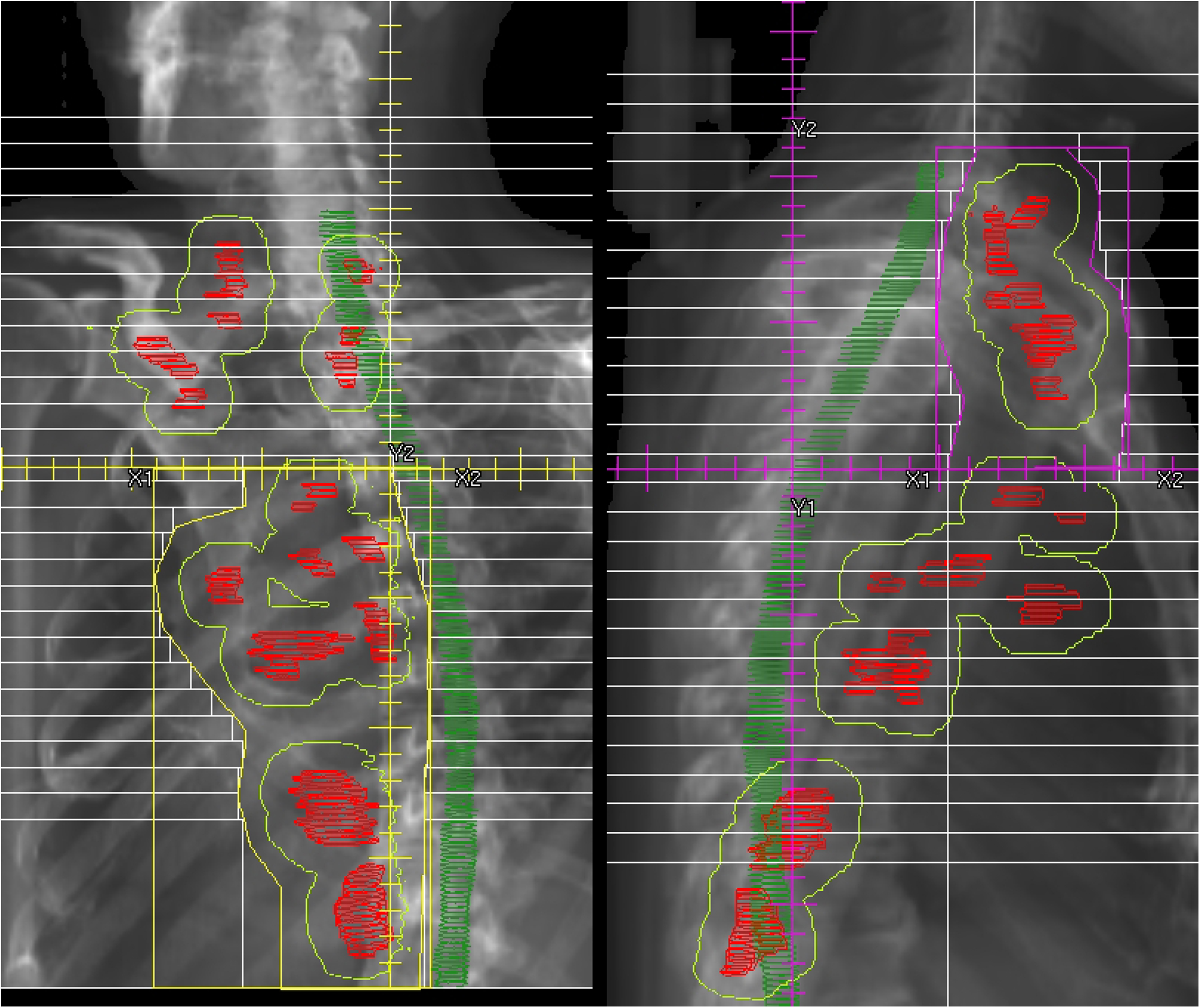

Supplement: Supplementary file 3 — Authors’ original file for figure 3 [file 40064_2014_1486_MOESM3_ESM.tif]
